# Decidual Stromal Cell Necroptosis Contributes to Polyinosinic-Polycytidylic Acid-Triggered Abnormal Murine Pregnancy

**DOI:** 10.3389/fimmu.2017.00916

**Published:** 2017-08-02

**Authors:** Shui-Xing Yu, Feng-Hua Zhou, Wei Chen, Gui-Mei Jiang, Chong-Tao Du, Gui-Qiu Hu, Zhen-Zhen Liu, Shi-Qing Yan, Jing-Min Gu, Xu-Ming Deng, Tong-Jun Lin, En-Kui Duan, Yong-Jun Yang

**Affiliations:** ^1^Key Laboratory of Zoonosis, Ministry of Education, College of Animal Medicine, Jilin University, Changchun, China; ^2^Department of Microbiology and Immunology, Dalhousie University, Halifax, NS, Canada; ^3^State Key Laboratory of Stem Cell and Reproductive Biology, Institute of Zoology, Chinese Academy of Sciences, Beijing, China

**Keywords:** abortion, mixed lineage kinase domain-like protein, necroptosis, pregnancy, polyinosinic-polycytidylic acid

## Abstract

Infectious agents can reach the placenta either *via* the maternal blood or by ascending the genito-urinary tract, and then initially colonizing the maternal decidua. Decidual stromal cells (DSCs) are the major cellular component of the decidua. Although DSCs at the maternal–fetal interface contribute to the regulation of immunity in pregnancy in the face of immunological and physiological challenges, the roles of these DSCs during viral infection remain ill defined. Here, we characterized the response of DSCs to a synthetic double-stranded RNA molecule, polyinosinic-polycytidylic acid [poly(I:C)], which is a mimic of viral infection. We demonstrated that both transfection of cells with poly(I:C) and addition of extracellular (non-transfected) poly(I:C) trigger the necroptosis of DSCs and that this response is dependent on RIG-I-like receptor/IPS-1 signaling and the toll-like receptor 3/TIR-domain-containing adapter-inducing interferon-β pathway, respectively. Furthermore, following poly(I:C) challenge, pregnant mixed lineage kinase domain-like protein-deficient mice had fewer necrotic cells in the mesometrial decidual layer, as well as milder pathological changes in the uterine unit, than did wild-type mice. Collectively, our results establish that necroptosis is a contributing factor in poly(I:C)-triggered abnormal pregnancy and thereby indicate a novel therapeutic strategy for reducing the severity of the adverse effects of viral infections in pregnancy.

## Introduction

Preterm birth or miscarriage is a common complication of pregnancy, and it does great harm to human health and hinders animal husbandry development. An estimated 15 million babies are born preterm annually, with infection being the most common cause (1). Infectious agents can reach the placenta either *via* the maternal blood or by ascending the genito-urinary tract and then initially colonizing the maternal decidua (2). Decidual stromal cells (DSCs) are the major cellular component of the decidua, and they play a critical role in embryo implantation and placentation (3, 4). In addition to their nutritive and endocrine functions, DSCs are also believed to have the potential to function as active members of the innate immune system, including by the production of cytokines and the modulation of uterine immune responses to sustain or compromise a normal pregnancy in the face of immunological and physiological challenges (5–8).

The innate immune response to a pathogen infection is initiated by the recognition of microbial products, termed pathogen-associated molecular patterns (PAMPs). PAMPs are recognized by a set of germ-line encoded pattern recognition receptors (PRRs), which allow detection of infection and initiation of the innate immune response (9–11). Several studies show that DSCs recognize and respond to bacterium-derived lipopolysaccharide or peptidoglycan through the upregulation of toll-like receptors (TLRs) or nod-like receptors during pregnancy (12–16). Binding to these PRRs usually results in the production of inflammatory cytokines through activation of the NF-κB signaling pathway, and this is detrimental to normal pregnancy.

During pregnancy, a wide variety of viral infections contribute to the occurrence of miscarriages and preterm birth. Moreover, decidual or endometrial stromal cells are permissive to common intrauterine viruses, including human cytomegalovirus and Zika virus (17–19), resulting in adverse pregnancy outcomes. Understanding of the response of DSCs to viral infection will help define new therapeutic strategies. Polyinosinic-polycytidylic acid [poly(I:C)], a synthetic analog of viral double-stranded RNA (dsRNA), is used to mimic viral infection (16). It has been demonstrated that the administration of poly(I:C) induces murine abortion (20, 21). However, little is known about the direct effects of dsRNA on DSCs during early pregnancy.

Recently, accumulating evidence has revealed that necroptosis, programmed necrosis of infected cells, plays a key role in removing the intracellular niche for microbial replication and exposing intracellular pathogens to extracellular immune surveillance (22, 23). During infection with influenza A virus, murine cytomegalovirus or vaccinia virus, necroptosis protects the infected animal (24–26). Although the death of the infected cells is an important host innate defense mechanism, excessive cell death is deleterious and pathological in host tissues. Examples also exist where increased necroptosis contributes to tissue injury and exacerbates viral disease (27, 28). Specifically, a recent study showed that necroptosis activation markers are differentially expressed in the placental tissues of preeclamptic women undergoing preterm labor (29).

In this study, we sought to characterize the response of DSCs to poly(I:C). We found that the administration of poly(I:C) induced necroptosis in DSCs. Transfected poly(I:C) induced DSC necroptosis in an RIG-I-like receptor (RLR)/IPS-1-dependent manner, while extracellular dsRNA induced necroptosis in a TLR3/TIR-domain-containing adapter-inducing interferon-β (TRIF)-dependent manner. Recently, necroptosis has been shown generally to be dependent on mixed lineage kinase domain-like protein (MLKL) (30). Using MLKL-deficient mice, we demonstrated that MLKL deficiency significantly improved pregnancy outcomes and reduced stromal cell death upon poly(I:C) challenge. Thus, our data provide important insight into the mechanisms underlying adverse pregnancy outcomes in response to infectious agents, such as dsRNA [poly(I:C)].

## Materials and Methods

### Reagents

The recombinant IFN-γ was purchased from Sino Biological Inc. (China). Lipofectamine 2000, Opti-MEM, DMEM/F12, and z-VAD-fmk (a pan-caspase inhibitor) were purchased from Invitrogen. Necrostatin-1 (Nec-1, a necroptosis inhibitor) was purchased from Tocris (UK). Poly(I:C) was purchased from Sigma-Aldrich. Receptor-interacting serine/threonine-protein kinase 3 (RIPK3) antibody was purchased from Abgent (USA). Phosphorylated MLKL antibody was purchased from Abcam (USA).

### Mice and Cells

TIR-domain-containing adapter-inducing interferon-β KO mice, on a C57BL/6 background, were a gift from Dr. Tong-Jun Lin (Dalhousie University, Canada) (31). MLKL KO mice, on a C57BL/6 background, were a gift from Dr. Jia-Huai Han (Xiamen University, China) (30). Both genotypes have no obvious reproductive dysfunction, with normal fertility and litter sizes. Subsequently, these two strains of mice were backcrossed to C57BL/6 background for another eight generations, respectively. C57BL/6 (WT), TRIF KO, and MLKL KO mice were housed in a pathogen-free facility and the animal studies were conducted according to experimental practices and standards approved by the Animal Welfare and Research Ethics Committee at Jilin University (No. 20150601). Uterine DSCs were isolated as described previously (32). Briefly, uterine horns from day 7.5 pregnant mice were split longitudinally and digested in HBSS containing 1% (w/v) trypsin (Amresco, USA) and 6 mg/ml dispase (Roche Diagnostics, USA). The digested uteri were shaken gently to dislodge sheets of luminal epithelial cells. The remaining tissues were rinsed three times with HBSS (Sigma-Aldrich, USA) and incubated in HBSS containing 0.15 mg/ml collagenase I (Invitrogen, USA) at 37°C for 30 min, followed by vigorously shaking until supernatants became turbid. The supernatants were then passed through a 70-μm wire gauze filter to eliminate epithelial sheets and centrifuged. The cell pellets were washed twice with HBSS and resuspended in complete medium consisting of DMEM/F12 media (Sigma-Aldrich) with 10% charcoal-treated fetal bovine serum (Invitrogen). Cells were plated onto 35-mm culture dishes at a concentration of 1 × 10^6^ cells/dish or 2 × 10^5^ cells/well for 24-well culture plates. After an initial culture for 30 min, the isolated stromal cells were further cultured in fresh medium with 2% cFBS. Subsequently, the cultured cells were identified by immunofluorescence staining for cytokeratin 8 or vimentin.

### Real-time Quantitative PCR

Total RNA was extracted from DSCs using TRI-reagent (Sigma-Aldrich) according to the manufacturer’s instruction. Two micrograms of total RNA were reverse-transcribed into cDNA using standard methods. Quantitative PCR assays were performed using SYBR Green on ABI Prism 7500 sequence detection system (Applied Biosystems). The following primers were used: GAPDH sense 5′-CACCCCAGCAAGGACACTGAGCAAG-3′, antisense 5′-GGGGGTCTGGGATGGAAATTGTGAG-3′. IFN-β sense 5′-ACTGCCTTTGCCATCCAAGA-3′, antisense 5′-CACTGTCTGCTGGTGGAGTT-3′. IPS-1 sense 5′-TCGGGACACCCAGTCATCTT-3′, antisense 5′-GA AACCGCAGCAGGAAAGTC-3′. Melanoma differentiation-associated protein 5 (MDA5) sense 5′-CAGTGACCTGGGATAAGGATGT-3′, antisense 5′-AGAAGA -GAAGGCAGAAGAAGCA-3′. RIG-I sense 5′-AAGCCAGAGACCAAGACCATTC-3′, antisense 5′-GAGCGTCATTCCTGTTGCCC-3′. TLR3 sense 5′-GCAAAGAAGATAAAGCGAGTTTCAC-3′, antisense 5′-GATAGAGAACAGGTGCGTCAAC-3′. Reactions were run using the manufacturer’s recommended cycling parameters of 50°C for 2 min, 95°C for 10 min, 40 cycles of 95°C for 15 s, and 60°C for 1 min. The relative expression of target transcripts in each sample was normalized to GAPDH according to the ΔCt method.

### RNAi and Cell Transfection

Three pairs of siRNAs against IPS-1 were designed by GenePharma Technologies (China). Their sequences were as following. IPS-1 1163 sense 5′-GCCACCUGUUUCAGUACUATT-3′, antisense 5′-UAGUACUGAAACAGGUGGCTT-3′. IPS-1 1221 sense 5′-CCAGAUUGGUCCCAGUAAATT-3′, antisense 5′-UUUACUGGGACCAAUCUGGTT-3′. IPS-1 1487 sense 5′-GGACCAAAUAGCAGUAUCATT-3′, antisense 5′-UGAUACUGCUAUUUGGUCCTT-3′. The following non-specific siRNAs were used as control. Scramble sense 5′-UUCUCCGAACGUGUCACGUTT-3′, antisense 5′-ACGUGACACGUUCGGAGAATT-3′. Cells were transfected with the siRNAs using the Lipofectamine 2000 (Invitrogen) according to the manufacturer’s instructions after cells were grown to 60% confluence.

### Annexin V/Propidium Iodide Staining

Phosphatidylserine exposure to the outer cell membrane was quantified by annexin V/PI staining followed by FACS analysis. Cells were stimulated for 24 h at 37°C with poly(I:C) in the presence or absence of z-VAD-fmk (25 µM) or Nec-1 (25 µM) as indicated. Annexin V/PI staining was performed according to the manufacturer’s instructions. All samples were assayed by a FACSAria flow cytometer (BD Biosciences) and the acquired data were further analyzed using FCS Express (*De Novo* Software).

### 3 -(4,5-Dimethylthiazol-2-yl)-2,5-Diphenyl Tetrazolium Bromide (MTT) Assay

Cell viability was investigated using an MTT (Sigma-Aldrich) assay. The cells were suspended in 0.1 ml of medium at a concentration of 6,000 cells/well and incubated overnight in 96-well plates. After stimulation by poly(I:C), the cells were incubated for 4 h with 0.8 mg/ml of MTT. Absorbance at 570 nm was measured using a microplate reader (BioTek, USA). Results were analyzed and presented as percentage of the control values.

### Cytokine Release Analysis

Mouse DSCs were seeded on 24-well plates and treated with indicated stimuli. Cell-free supernatants were collected and subjected to analysis with DuoSet ELISA kits (R&D Systems, USA) following the manufacturer’s instructions.

### Western Blotting

The cells were cultured in 12-well plates and treated with indicated stimuli. Cells were harvested at different time points and lysed in RIPA buffer. Total cell lysates were separated by SDS-PAGE, and transferred onto PVDF membrane, and western blotting was performed with the appropriate antibodies. The proteins were visualized by enhanced chemiluminescence detection reagent (Millipore, USA).

### Transmission Electron Microscopy

After stimulation, cells were digested with 0.25% trypsin and suspended at a density of 1.0 × 10^6^/ml. Then 3% glutaraldehyde was added for 3 h at 4°C for fixation. Ultrathin sections (100 nm) were prepared, stained with uranyl acetate and lead citrate, and examined under an electron transmission microscope.

### Histology and TUNEL Staining

For histology, uterine tissue was fixed in 4% neutral buffered formalin and sections were stained with hematoxylin and eosin (H&E) to examine morphologic changes. TUNEL staining was performed using a fluorescent detection kit (Roche Diagnostics), following the manufacturer’s instructions.

### Immunohistochemistry

After deparaffinization, the uterine unit sections were hydrated and epitope retrieval was performed. The tissue sections were stained using phosphorylated MLKL antibody, followed by secondary HRP-labeled polymer with DAB staining and hematoxylin counterstaining.

### Statistical Analysis

All values are expressed as mean ± SD. Differences between mean values of normally distributed data were assessed with one-way ANOVA (Dunnett’s *t*-test) and two-tailed Student’s *t*-test. *P < 0.05 and **P < 0.01 compared with control group.

## Results

### Transfected Poly(I:C) Induces Necroptosis in DSCs

**Figure 1:**
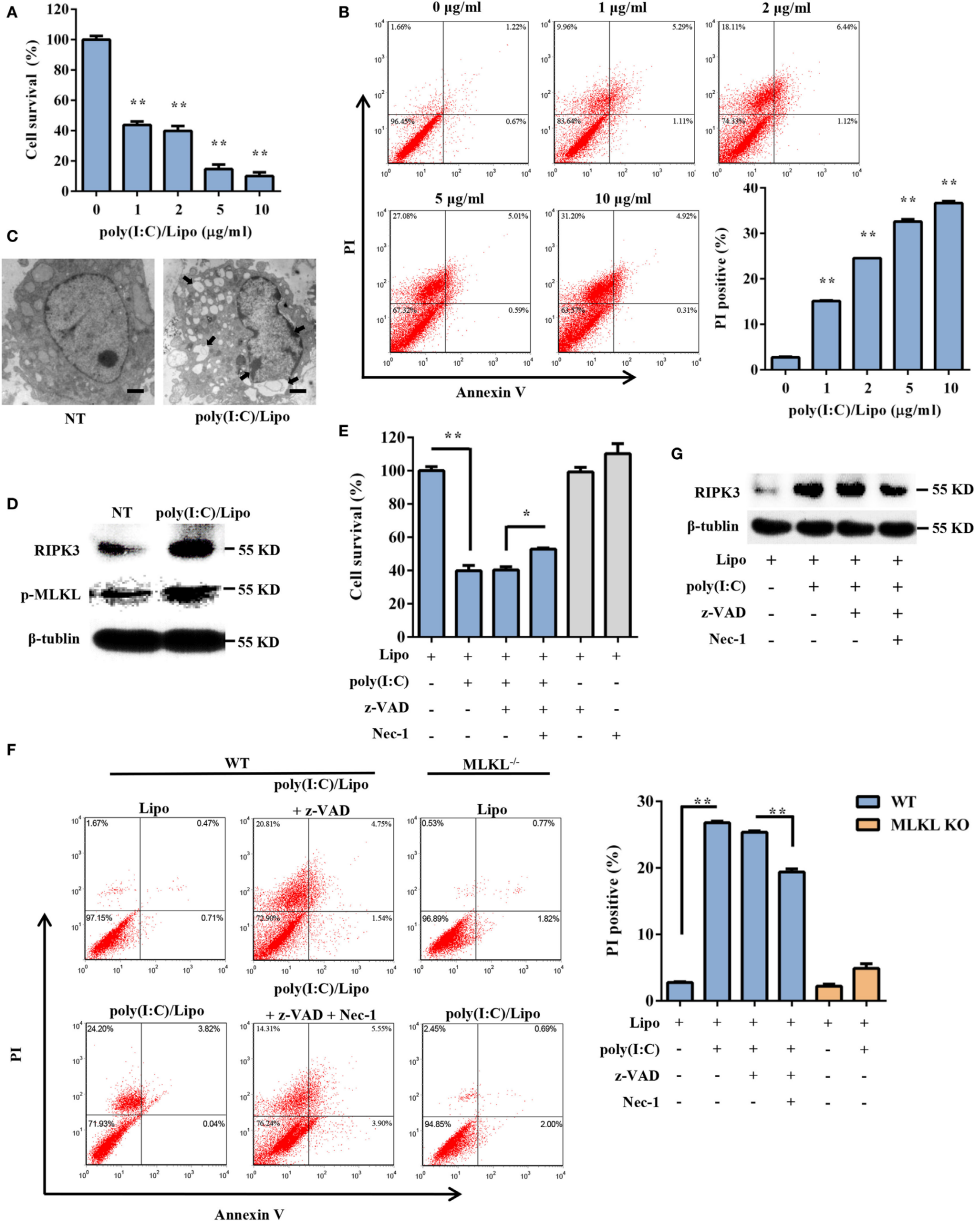
Transfection with polyinosinic-polycytidylic acid [poly(I:C)] induces necroptotic cell death in decidual stromal cells (DSCs). **(A)** DSCs were seeded at 6,000 cells/well into a 96-well plate and were then treated with different doses of poly(I:C)/Lipo complex. The viability of the DSCs was determined by a 3-(4,5-dimethylthiazol-2-yl)-2,5-diphenyl tetrazolium bromide (MTT) assay 24 h after transfection. Data represent the mean ± SEM of three independent experiments. **(B)** Approximately 2 × 10^5^ DSCs were double stained with annexin-V–fluorescein isothiocyanate and PI after transfection. FACS scatter plots representative of three independent experiments are shown. The PI incorporation (%) was plotted after the statistical analysis. **(C)** The morphological features of the dead cells were examined by transmission electron microscope. The scale bar represents 5 µm. Three independent experiments were performed. **(D)** Receptor-interacting serine/threonine-protein kinase 3 (RIPK3) and phosphorylated mixed lineage kinase domain-like protein (MLKL) expression in the DSCs was detected by Western blotting. β-tubulin was used as a loading control. **(E–G)** DSCs were treated for 24 h with 2 µg/ml of poly(I:C)/Lipo complex in the presence or absence of z-VAD-fmk and necrostatin-1 (Nec-1). **(E)** The DSC viability was determined by an MTT assay. **(F)** Cell death was determined by annexin V/PI staining and FACS analysis. FACS scatter plots representative of three independent experiments are shown. The PI incorporation (%) was plotted after the statistical analysis. **(G)** The cell lysates were prepared and then subjected to Western blot analysis for evaluation of the RIPK3 protein. Data are representative of two separate experiments with similar results. *P < 0.05 and **P < 0.01, compared with the control group.

To explore the possible involvement of DSCs in response to dsRNA virus infection, the cell viability of primary DSCs was examined after the administration of a synthetic dsRNA analog, poly(I:C), complexed with Lipofectamine 2000 (Lipo). Twenty-four hours after stimulation, DSC viability was determined using the MTT assay. Transfection of DSCs with poly(I:C) resulted in dose-dependent cell death (Figure 1A). To further characterize the poly(I:C)-triggered DSC death, 24 h after stimulation, the DSCs were stained with annexin V/PI and were subjected to FACS analysis. The cell population that was positive for both annexin V and PI was elevated. Specifically, transfected poly(I:C) induced substantial cellular necrosis, as shown by a significantly higher PI uptake (%) by these cells than by control cells (Figure 1B). Moreover, transmission electron microscope analysis demonstrated that poly(I:C)-stimulated DSCs exhibited an increased cell volume, swelling of organelles, and a translucent cytoplasm (Figure 1C), suggesting necrotic cell death.

Necroptosis is a programmed form of necrosis, mediated by RIPK3 and MLKL. We observed that poly(I:C) transfection induced a strong increase in the expression of RIPK3 and phosphorylated MLKL (Figure 1D). We next examined whether pharmacologically inhibiting necroptosis attenuated the cell death mediated by poly(I:C). Indeed, poly(I:C)-mediated necrosis was significantly inhibited by Nec-1, a specific inhibitor of the kinase activity of RIPK1 (Figures 1E,G). The PI uptake and the MTT assay supported this finding. We also observed that poly(I:C)-induced cell death was insensitive to the pan-caspase inhibitor z-VAD-fmk. Moreover, compared to the WT cells treated with poly(I:C), significantly fewer MLKL-deficient cells that were treated with poly(I:C) underwent cell death (Figure 1F). Taken together, these data demonstrated that transfected dsRNA-induced necroptosis in DSCs.

### Poly(I:C) Transfection-Induced Necroptosis Does Not Depend on the TLR3/TRIF Signaling Pathways

**Figure 2:**
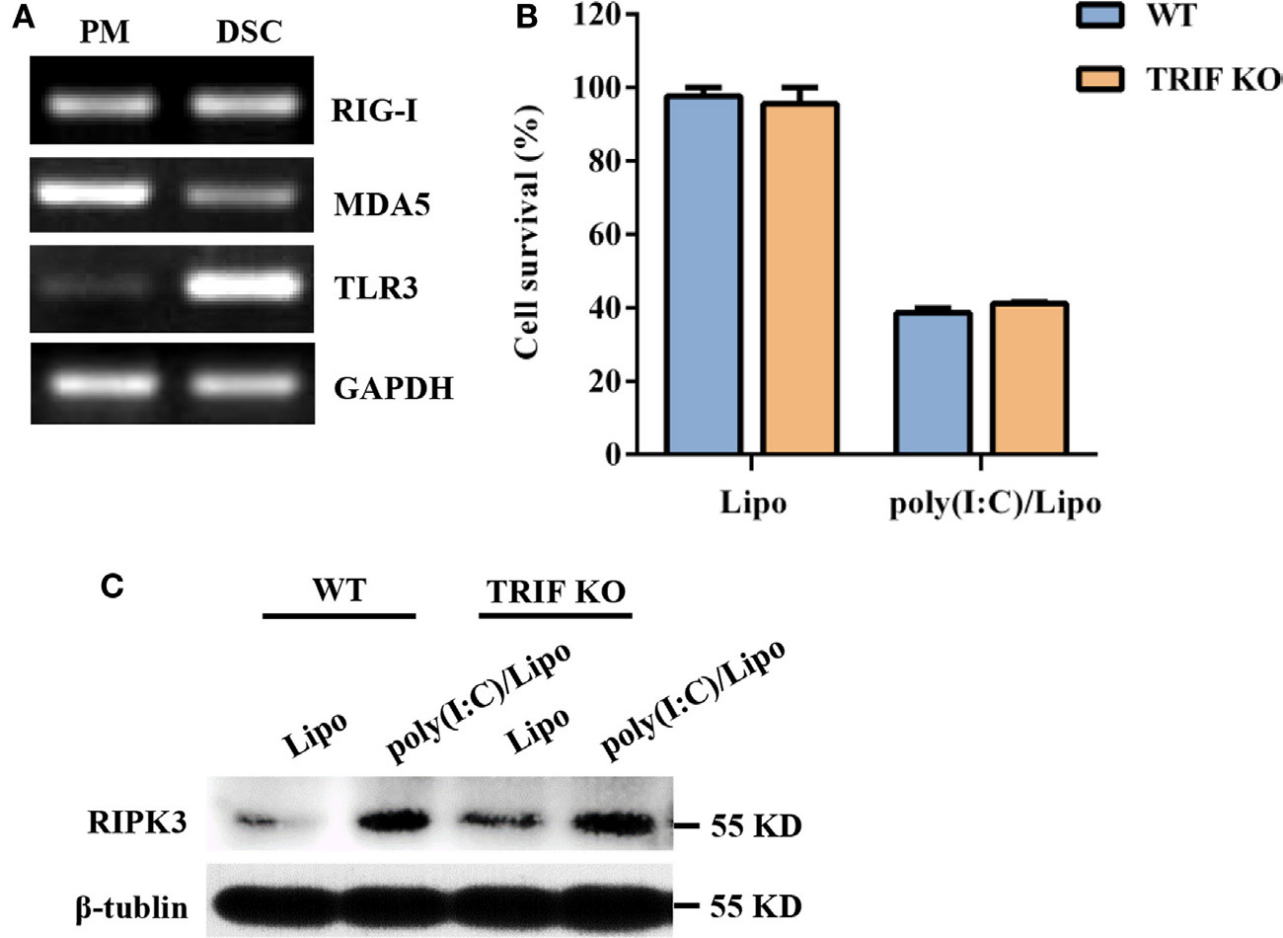
Polyinosinic-polycytidylic acid [poly(I:C)] transfection-induced decidual stromal cell (DSC) necroptosis does not depend on toll-like receptor (TLR) 3/TIR-domain-containing adapter-inducing interferon-β (TRIF) signaling. **(A)** Total RNA was extracted from peritoneal macrophages (PMs) and DSCs. The levels of RIG-I, melanoma differentiation-associated protein 5 (MDA5), and TLR3 mRNA expression were determined by RT-PCR. GAPDH is used as an internal control. **(B)** DSCs from WT and TRIF KO mice were seeded at 6,000 cells/well into a 96-well plate and were then treated with 2 μg/ml of the poly(I:C)/Lipo complex. The viability of the DSCs was determined by a 3-(4,5-dimethylthiazol-2-yl)-2,5-diphenyl tetrazolium bromide assay 24 h after transfection. Data represent the mean ± SEM of three independent experiments. **(C)** The cell lysates were prepared and were subjected to Western blot analysis for evaluation of the receptor-interacting serine/threonine-protein kinase 3 (RIPK3) protein. Data are representative of two separate experiments with similar results.

Double-stranded RNA is recognized by at least two types of PRRs: RIG-I-like receptors (RIG-I and MDA5) and TLR3. We first characterized the expression profiles of these PRR components in DSCs. Mouse peritoneal macrophages were used as a positive control. DSCs constitutively expressed RIG-I, MDA5, and TLR3 messenger RNA (Figure 2A). We next examined whether the poly(I:C)-induced necroptosis was dependent on the TLR3/TRIF signaling pathway. DSCs from WT and TRIF KO mice were treated with 2 μg/ml of the poly(I:C)/Lipo complex, and the MTT assay indicated that the decreased cell viability was comparable (Figure 2B). Furthermore, no obvious difference in RIPK3 expression was observed between TRIF KO DSCs and WT DSCs (Figure 2C). Thus, dsRNA transfection-induced necroptosis was not dependent on TLR3/TRIF signaling.

### Poly(I:C) Transfection Induces Necroptosis in an RLR/IPS-1-Dependent Manner

**Figure 3:**
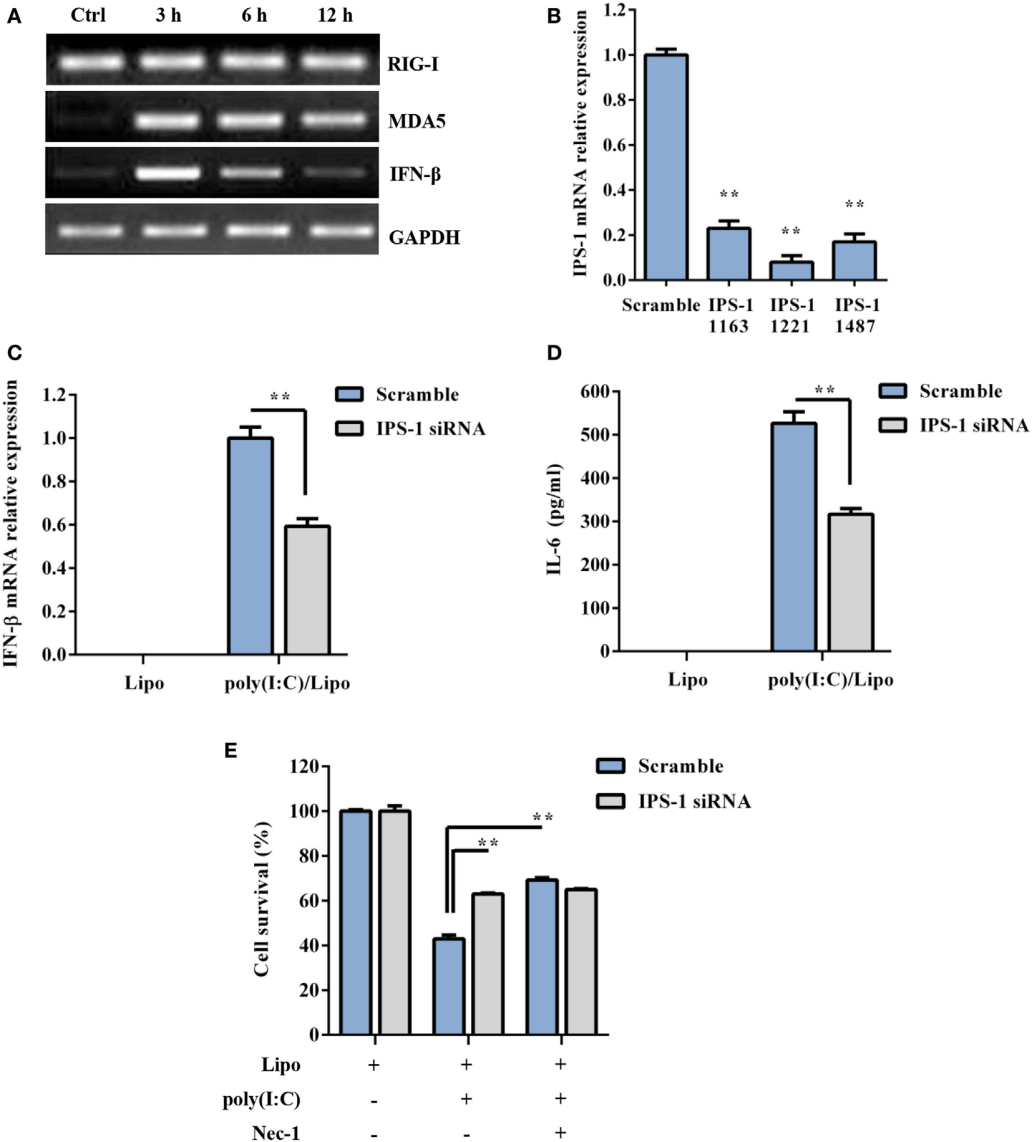
Polyinosinic-polycytidylic acid [poly(I:C)] transfection-induced decidual stromal cell (DSC) necroptosis depends on RLRs/IPS-1 signaling. **(A)** DSCs were treated with 2 µg/ml of the poly(I:C)/Lipo complex for 3, 6, and 12 h. The levels of IPS-1, RIG-I, and IFN-β mRNA were determined by semiquantitative RT-PCR. GAPDH was used as an internal control. **(B)** The cells were transfected with IPS-1 siRNAs (IPS-1 1163, IPS-1 1221, IPS-1 1487) or scrambled siRNA. IPS-1 mRNA expression was analyzed by quantitative RT-PCR. **(C,D)** The cells were transfected with IPS-1 1221 siRNA or scrambled siRNA and were then treated with 2 μg/ml of the poly(I:C)/Lipo complex. **(C)** IFN-β expression was analyzed by quantitative RT-PCR. **(D)** The concentration of IL-6 in the cell-free supernatants was measured by ELISA. **(E)** IPS-1 siRNA or scrambled siRNA-transfected cells were treated for 24 h with 2 μg/ml of the poly(I:C)/Lipo complex in the presence or absence of z-VAD-fmk and necrostatin-1 (Nec-1). The cell viability was determined by a 3-(4,5-dimethylthiazol-2-yl)-2,5-diphenyl tetrazolium bromide assay. Data represent mean ± SEM of three independent experiments. *P < 0.05 and **P < 0.01, compared with the control group.

RIG-I and MDA5 also recognize atypical RNAs associated with viral infection. They initiate signaling through the recruitment of an adaptor, IPS-1, and result in the activation of the transcription factors IRF3 and NF-κB for the induction of type I IFNs and inflammatory cytokines, respectively. As shown in Figure 3A, RIG-I was constitutively expressed, while MDA5 and IFN-β were inducible 3–12 h following poly(I:C) transfection. To determine whether RLR/IPS-1 signaling pathway is involved in poly(I:C)-induced DSC death, we employed an siRNA approach. The expression of IPS-1 mRNA was efficiently suppressed through the transfection of specific siRNAs (IPS-1 1163, IPS-1 1221, or IPS-1 1487) (Figure 3B). A decrease in IFN-β and IL-6 production, in response to poly(I:C) challenge, confirmed the silencing of IPS-1 (Figures 3C,D). IPS-1 knockdown significantly inhibited poly(I:C) transfection-induced cell death compared with scrambled siRNA (Figure 3E). Therefore, these findings suggested that cytoplasmic poly(I:C) induced necroptosis in DSCs in an RLR/IPS-1-dependent manner.

### Extracellular Poly(I:C) Induces Necroptosis in a TLR3/TRIF-Dependent Manner

**Figure 4:**
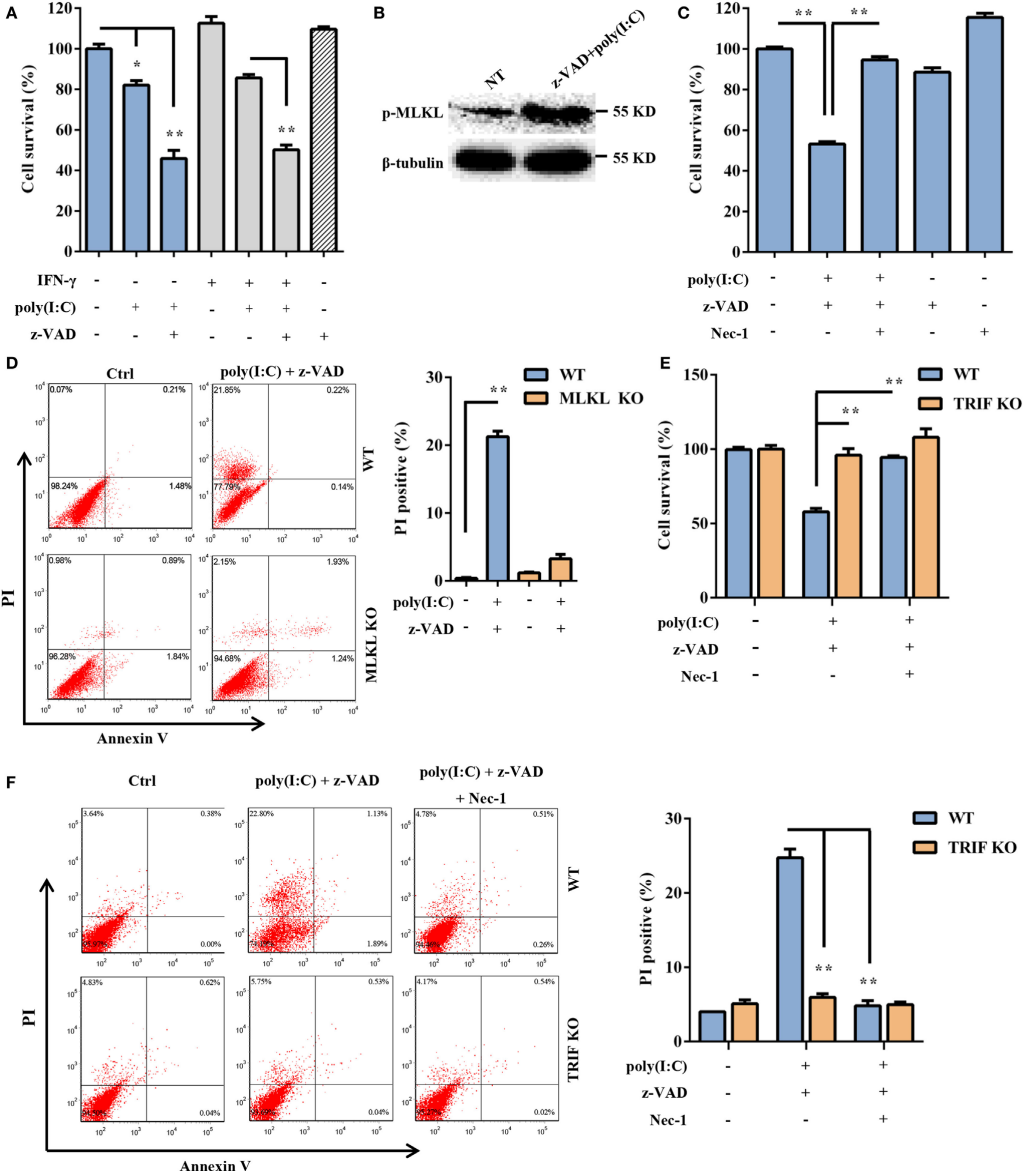
Extracellular polyinosinic-polycytidylic acid [poly(I:C)]-induced decidual stromal cell (DSC) necroptosis depends on the toll-like receptor 3/TIR-domain-containing adapter-inducing interferon-β (TRIF) pathway. **(A)** DSCs were treated for 24 h with 100 μg/ml of poly(I:C) and 20 ng/ml of IFN-γ in the presence or absence of z-VAD-fmk. Cell viability was determined by a 3-(4,5-dimethylthiazol-2-yl)-2,5-diphenyl tetrazolium bromide (MTT) assay. Data represent the mean ± SEM of three independent experiments. **(B)** Phosphorylated mixed lineage kinase domain-like protein (MLKL), in the DSCs, was detected by Western blotting. β-tubulin was used as a loading control. **(C)** The cells were treated for 24 h with poly(I:C) in the presence or absence of z-VAD-fmk and necrostatin-1 (Nec-1). The cell viability was determined by an MTT assay. The data are the mean ± SEM of four independent experiments. **(D)** Cell death was determined by annexin V/PI staining and FACS analysis. FACS scatter plots representative of three independent experiments are shown. The PI incorporation (%) was plotted after the statistical analysis. **(E,F)** DSCs from WT or TRIF KO mice were treated with poly(I:C) in the presence or absence of z-VAD-fmk and Nec-1. **(E)** The cell viability was determined by an MTT assay. **(F)** Cell death was determined by annexin V/PI staining and FACS analysis. FACS scatter plots representative of three independent experiments are shown. The cell death index (%) was plotted after the statistical analysis. *P < 0.05 and **P < 0.01, compared with the control group.

Subsequently, we assessed whether extracellular dsRNA-induced DSC death. DSCs were stimulated with free poly(I:C) for 24 h and were subjected to a cell viability analysis using the MTT assay. The cytotoxic effect of non-transfected poly(I:C) was not as strong as that of transfected poly(I:C) (Figure 4A). It has been shown that some stimuli can elicit necrotic cell death when caspase activation is impaired. As expected, non-transfected poly(I:C) significantly induced DSC death and phosphorylated MLKL expression in the presence of z-VAD-fmk, which alone was not toxic to the target cells (Figures 4A,B). Poly(I:C) plus IFN-γ was included as the positive control (33, 34). In the presence of Nec-1, non-transfected poly(I:C) plus z-VAD-fmk-induced cell death was significantly inhibited (Figure 4C). Moreover, we observed that non-transfected poly(I:C) plus z-VAD-fmk-induced cell death was significantly inhibited by MLKL deficiency (Figure 4D). These results indicated that extracellular poly(I:C) also induced DSC necroptosis. TLR3 is exclusively expressed in the endoplasmic reticulum in the unstimulated condition and is delivered to endosomes, where it recognizes dsRNA and engages with the adaptor TRIF, further activating IRF3 and NF-κB and mediating the transcriptional activation of proinflammatory cytokines and type I IFNs. Therefore, to determine whether the TLR3/TRIF signaling pathway is involved in extracellular poly(I:C)-induced DSC necroptosis, primary DSCs from WT mice and TRIF KO mice were treated with poly(I:C) in the presence of z-VAD-fmk and Nec-1. The MTT assay indicated that poly(I:C) complexed with z-VAD-fmk induced little cell death in TRIF KO DSCs (Figure 4E). FACS analysis showed a similar result (Figure 4F). Together, these results demonstrated that extracellular dsRNA-induced necroptosis in a TLR3/TRIF-dependent manner.

### Poly(I:C) Strongly Triggers Abnormal Pregnancy and Stromal Cell Death in WT Mice Compared with MLKL KO Mice

**Figure 5:**
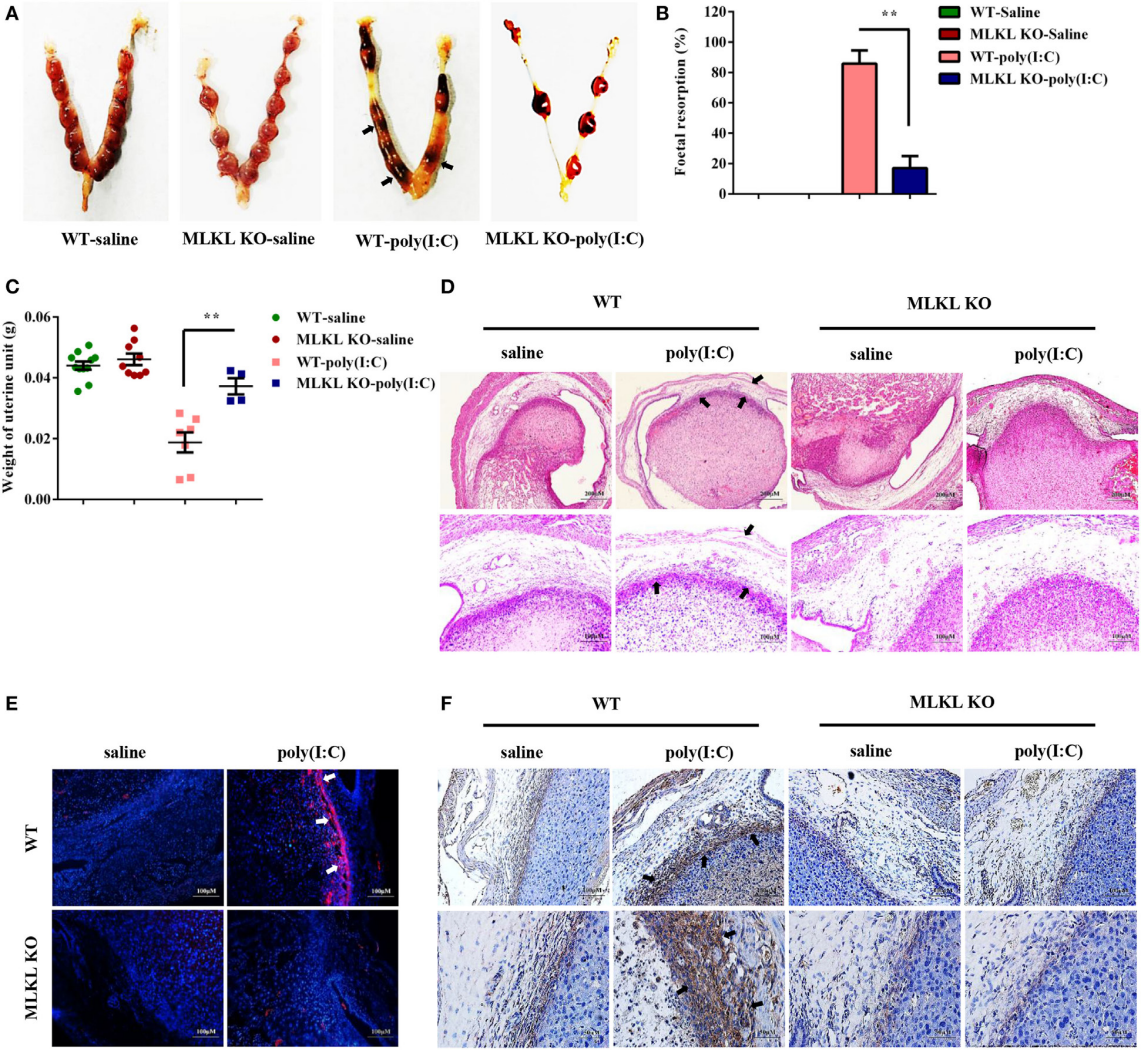
Polyinosinic-polycytidylic acid [poly(I:C)] strongly triggers abnormal pregnancy and stromal cell death in WT mice compared with mixed lineage kinase domain-like protein (MLKL) KO mice. Female WT and MLKL KO mice were intraperitoneally challenged with poly(I:C) (30 mg/kg) at gestation day 7.5 and then euthanized on gestation day 9.5. Pregnant control mice were injected with saline. **(A)** Representative uterine horns are shown. **(B)** Fetal resorption was assessed by inspection of the uterus. A summary of three independent experiments with eight mice per group is shown. **(C)** The uterine unit weights were determined. **(D)** The uterine unit tissue sections were stained with hematoxylin and eosin. Representative staining sections are shown. **(E)** The uterine unit tissue sections were stained with TUNEL. DAPI was used for nucleus staining. The data are representative. **(F)** The uterine unit tissue sections were stained using a phosphorylated MLKL antibody. The data are representative. **P < 0.01, compared with the control group.

To determine whether the necroptotic signal mediated dsRNA-triggered abnormal murine pregnancy, WT and MLKL KO mice were intraperitoneally administered with 30 mg/ml of poly(I:C) during early pregnancy (7.5 days). We found that the administration of poly(I:C) caused a higher frequency of fetal resorption in WT mice than in MLKL KO mice (Figures 5A,B). To further assess the severity of the abnormal pregnancy, the uterine unit weights of the WT and MLKL KO mice were measured on postinjection day 2. We observed that the WT mice exhibited severe uterus hemorrhage and uterine unit weight loss (Figure 5C). These clinical assessments were validated by the histological examination of the uterine unit using H&E staining (Figure 5D). The potentially maladaptive responses to poly(I:C) observed in the WT uterine unit included the exaggerated compression of the mesometrial decidua and fetal dysplasia compared with the MLKL KO mice. Moreover, TUNEL staining of the histological sections of the uterine unit showed that the WT mice had many more TUNEL-positive cells in the mesometrial decidual layer, whereas the MLKL KO mice showed only a slight increase (Figure 5E). In addition, immunohistochemical analyses showed that the positive phosphorylated MLKL signal exhibited features that were similar to TUNEL staining upon stimulation with poly(I:C) (Figure 5F). Collectively, these results revealed that DSC necroptosis played a critical role in poly(I:C)-induced abnormal murine pregnancy.

## Discussion

As the major cellular component of maternal decidua, DSCs may be exposed to various viruses, which results in adverse pregnancy outcomes. DSCs also act as active members of the innate immune system and are armed with a wide variety of sensors to recognize the PAMPs of invading viruses. Immune sensing of virus-derived nucleic acids has emerged as a central component of the innate response to viral intrusion. Cell death is one of the host innate immune response that is induced in response to pathogen infection or PAMP challenge. This observation prompted us to examine whether dsRNA triggers DSC death. First, DSCs were transfected with a concentration gradient of poly(I:C). We observed that transfection with poly(I:C) induced DSC death in a dose-dependent manner. The morphological characteristics of DSC death further implied that dsRNA induces a necrosis-like cell death. This observation suggests a potential role for DSC death in dsRNA viral infection during pregnancy.

Host cells can die in a variety of ways, such as apoptosis, necroptosis, autophagy, pyroptosis, necrosis, and oncosis. Therefore, it is rational to explore the manner of dsRNA-induced DSC death. Interestingly, we observed that poly(I:C) induced RIPK3 expression and phosphorylated MLKL. In addition to acting as a regulator of inflammation, RIPK3 also plays an essential role in the activation of necroptosis (24, 35). The activity of the protein kinase RIPK3 determines whether cells die by necroptosis or apoptosis. Indeed, we observed that the presence of z-VAD-fmk did not reverse dsRNA-induced cell death but that Nec-1 dramatically inhibited cell death induction. Furthermore, transfected poly(I:C)-induced cell death was significantly suppressed by MLKL deficiency. Thus, these results support the finding that poly(I:C) triggers DSC necroptosis rather than apoptosis.

Pattern recognition receptors, such as TLR3 and RLRs, recognize viral dsRNA and trigger an antiviral response in various cell types, especially immune cells (36–38). TLR3 is a transmembrane receptor with leucine-rich repeats and a cytoplasmic toll/IL-1 receptor homology (TIR) domain (36, 39). Upon encountering its cognate ligand, TLR3 activates intracellular signaling cascades by recruiting the TIR-domain-containing adaptor TRIF and then activating the transcription factors IRF3 and NF-κB, which mediate type I IFN and inflammatory cytokine production (40, 41). The RLR family members, including RIG-I and MDA5, are RNA helicases that share a homologous DExD/H box (42). The helicase domains of RIG-I and MDA5 recognize dsRNA, and their CARDs are responsible for signaling by interacting with a CARD-containing adaptor called IPS-1. This interaction results in the activation of IRF3, IRF7, and NF-κB and thereby the induction of type I IFNs and proinflammatory cytokines. TLR3, RIG-I, and MDA5 are highly expressed or induced in DSCs, suggesting that both pathways could potentially mediate poly(I:C)-triggered DSC necroptosis. However, transfected poly(I:C) induced necroptosis to a similar extent in the WT and TRIF KO DSCs. Thus, transfected poly(I:C) induces DSC necroptosis in a TLR3/TRIF-independent manner. Conversely, the knockdown of IPS-1 markedly inhibited transfected poly(I:C)-induced DSCs death, indicating that intracellular poly(I:C) triggers DSC necroptosis in a manner that is dependent on the RLR/IPS-1 signaling pathway. In addition to transfected poly(I:C), non-transfected poly(I:C) also induced DSC necroptosis in the presence of z-VAD-fmk. Non-transfected poly(I:C) did not induce DSC necroptosis upon TRIF deficiency, which suggested that extracellular poly(I:C)-triggered DSC necroptosis is dependent on the TLR3/TRIF signaling pathway. TLR3 is exclusively assembled in the endoplasmic reticulum in the unstimulated condition and is delivered to endosomes by the transmembrane protein UNC93B1, where it recognizes dsRNA and directly recruits TRIF to its TIR domain to initiate signaling (43). Following the uptake of extracellular dsRNA into the endosome, TLR3/TRIF-mediated necroptotic signaling is activated. RIG-I and MDA5 recognize cytoplasmic dsRNA, which is delivered by dsRNA transfection or produced by viral intracellular replication. Therefore, distinct intracellular and extracellular dsRNA-activated signaling pathways lead to DSC necroptosis.

Very recently, the indispensable role of MLKL in necroptosis was demonstrated using MLKL knockout mice (30). Once activated, RIPK3 phosphorylates the substrate MLKL and triggers its oligomerization, both of which are necessary and sufficient for necroptotic cell death (28, 44–46). To explore whether the necroptotic signal is involved in the regulation of dsRNA-triggered abnormal pregnancy, pregnant MLKL knockout mice were subjected to poly(I:C)-induced abortion. The pattern of the uterus hemorrhage, fetal loss, and resorption observed in the WT mice but not in the MLKL KO mice following poly(I:C) administration suggested that the necroptotic signal plays a maladaptive role in maintaining normal pregnancy. To further characterize the role of DSC necroptosis in dsRNA-triggered abnormal pregnancy, TUNEL staining and a histological examination of the uterine unit were carried out. The results showed that the mesometrial decidua was greatly compressed, and there was severe fetal dysplasia in the WT mice compared with MLKL KO mice. Furthermore, TUNEL staining and phosphorylated MLKL positive cells were found largely in the mesometrial decidual layer of the WT mice in contrast to MLKL KO mice. Thus, these observations suggest that marked DSC necroptosis is triggered by dsRNA, and, in the setting of murine pregnancy, contributes to adverse outcomes.

In summary, our findings demonstrated that poly(I:C) induced DSC necroptosis and that DSC necroptosis mediated dsRNA-triggered abnormal murine pregnancy. Thus, intervention in the signaling leading to DSC necroptosis may aid in efforts to prevent and treat dsRNA-related abnormal pregnancy. These findings also contribute to better defining the possible role of DSC necroptosis in pregnancy-associated diseases.

## Ethics Statement

All animal studies were conducted according to experimental practices and standards approved by the Animal Welfare and Research Ethics Committee at Jilin University (No. 20150601).

## Author Contributions

YSX, ZFH, CW, and YYJ designed experiments. YSX, ZFH, CW, JGM, DCT, HGQ, LZZ, YSQ, and GJM performed the experiments and analyzed the data. YSX and YYJ wrote the manuscript. DXM, LTJ, and DEK revised the manuscript. All authors read and approved the final manuscript.

## Conflict of Interest Statement

The authors declare that the research was conducted in the absence of any commercial or financial relationships that could be construed as a potential conflict of interest.

## Abbreviations

DSCs, decidual stromal cells; dsRNA, double-stranded RNA; MLKL, mixed lineage kinase domain-like protein; RIPK1/3, receptor-interacting serine/threonine-protein kinase 1/3; TRIF, TIR-domain-containing adapter-inducing interferon-β; IPS-1, IFN-β promoter stimulator 1; MDA5, melanoma differentiation-associated protein 5; RLRs, RIG-I-like receptors; TLRs, toll-like receptors; NLRs, nod-like receptors; PAMPs, pathogen-associated molecular patterns; PRRs, pattern recognition receptors; LPS, lipopolysaccharide; HCMV, human cytomegalovirus; MTT, 3-(4,5-dimethylthiazol-2-yl)-2,5-diphenyl tetrazolium bromide; TEM, transmission electron microscope; Nec-1, necrostatin-1; PM, peritoneal macrophage; H&E, hematoxylin and eosin.
